# Evaluating LDL-C control in Indian acute coronary syndrome (ACS) patients- A retrospective real-world study LDL-C control in ACS

**DOI:** 10.1016/j.ijcrp.2023.200210

**Published:** 2023-09-16

**Authors:** Madhur Jain, Rahul Sawant, Hitanshu Panchal, Anand S, Anupam Jena, Rahul Gupta, Karthik Kumar, Rajagopal Jambunathan, Sunil Modi, Ajit Mullasari, Nakul Sinha, Kimi Shetty, Pallavi Kawatra

**Affiliations:** a Dr Madhu Jain's Clinic, New Delhi, India; b Hridaymitra Cardia Clinic, Pune, India; c Savita Super Speciality Hospital, Vadodara, India; d Kauvery Heart City, Tamil Nadu, India; e KIMS Hospital, Bhubaneswar, India; f Cardium Clinic, Mumbai, India; g Smile Health Clinic, Coimbatore, India; h Cauvery Heart and Multi-Specialty Hospital, Mysore, India; i Indraprasatha Apollo Hospital, New Delhi, India; j Madras Medical Mission Hospital, Chennai, India; k Medanta Hospital, Lucknow, India; l Medical Lead, Novartis Healthcare Private Limited, Mumbai, India; m Franchise Medical Head, Novartis Healthcare Private Limited, Mumbai, India

**Keywords:** LDL-C, Statins, Acute coronary syndrome, India, Index date, LLT

## Abstract

**Background:**

Low-density lipoprotein-cholesterol (LDL-C) is an independent risk factor for atherosclerotic cardiovascular disease (ASCVD) progression. Although lipid lowering therapies remain the cornerstone of secondary ACSVD prevention, there exists residual dyslipidemia. The current study aimed to evaluate the real-world experience related to the treatment patterns and LDL-C control in Indian Acute Coronary Syndrome (ACS) patients.

**Methods:**

This was a real-world, descriptive, retrospective, observational, and multicentric study conducted across India. The data was collected for 1 year following the ACS event. The change in the levels of LDL-C from the baseline to the follow-up visits and the control of LDL-C, the change in lipid profile, lipoprotein levels, treatment patterns for lipid-lowering, and tolerability of existing treatments were evaluated.

**Results:**

Overall, 575 patients were included from 11 centers across India. The mean age of the patients was 52.92 years, with male predominance (76.35%). Although there was a significant reduction in the mean levels of LDL-C from the baseline [(122.64 ± 42.01 mg/dl to 74.41 ± 26.45 mg/dl (p < 0.001)], it was observed that despite high-intensity statin therapy, only 20.87% patients managed to achieve target LDL-C of <55 mg/dL and 55.65% were unable to reach LDL-C levels of <70 mg/dl one year after the event. Six patients reported adverse events without treatment discontinuation.

**Conclusion:**

The majority of the patients received high-intensity statins and did not attain target LDL-C levels, suggesting LDL-C control after an ACS event requires management with novel therapies having better efficacy as recommended by international and national guidelines.

## Abbreviations

ACC/AHA: American College of Cardiology/American Heart Association
ACS: Acute Coronary Syndrome
Apo (B): Apolipoprotein B
ARIC: Atherosclerosis Risk in Communities
ASCVD: Atherosclerotic Cardiovascular Disease
BMI: Body Mass Index
BP: Blood Pressure
CABG: Coronary Artery Bypass Graft
CEPHEUS: Centralized Pan-Middle East Survey on the Under-Treatment of Hypercholesterolemia
CHD: Coronary Heart Disease
CKD: Chronic Kidney Disease
CTRI: Clinical Trial Registry of India
DYSIS: Dyslipidemia International Study
EECP: Enhanced External Counter Pulsation
EMR: Electronic Medical Records
ESC/EAS: European Society of Cardiology/European Atherosclerosis Society
FH: Familial Hypercholesteremia
GCP: Good Clinical Practice
HDL: High-Density Lipoprotein
ICH-GCP: International Council for Harmonisation-Good Clinical Practice
LAI: Lipid Association of India
LDL-C: Low-Density Lipoprotein-Cholesterol
LDL: Low Density Lipoprotein
LLT: Lipid-Lowering Therapies
Lp(a): Lipoprotein a
MI: Myocardial Infarction
NHDL: Non-High-Density Lipoprotein
NSTEMI: Non-ST (-segment) Elevation Myocardial Infarction
OR: Odds Ratio
PAR: Population Attributable Risk
PCSK9: Proprotein Convertase Subtilisin/Kexin Type 9
PSVT: Paroxysmal Supraventricular Tachycardia
PTB: Pulmonary Tuberculosis
STEMI: ST segment Elevation Myocardial Infarction
T2DM: Type 2 Diabetes Mellitus
TC: Total Cholesterol
TG: Triglycerides
UA: Unstable Angina
VLDL: Very Low-Density Lipoprotein

## 1 Introduction

In India, 1 in 4 deaths has been reported to be because of cardiovascular diseases with acute coronary syndrome (ACS) and stroke responsible for >80% of this burden [1]. ACS refers to a group of conditions that include unstable angina (UA), non-ST (-segment) elevation myocardial infarction MI (NSTEMI), and ST (-segment) elevation MI (STEMI), which ensues from the occlusion of coronary arteries [2]. It is a manifestation of coronary heart disease, responsible for one-third of total deaths in people older than 35 years of age [3]. The pathogenesis of ACS involves reduced blood flow to a part of the heart musculature, which is usually secondary to atherosclerotic plaque rupture and thrombus formation [4].

Low-density lipoprotein-cholesterol (LDL-C) has been established as a critical cardiovascular disease marker to estimate the risk of ACS, as several studies evidenced that reduction of LDL-C level prevents cardiovascular outcomes [5,6]. In a large case-control INTERHEART study, it was identified that among all the modifiable risk factors, abnormal lipid levels were found to be associated with the highest population attributable risk (PAR 49.2%) for the occurrence of myocardial infarction. The most important risk factor identified across all geographic regions was ApoB/Apo A1 ratio (OR 4.73, 99% CI 3.93–5.69) [7]. In the ARIC study, it was identified that, every 39 mg/dL incremental increase in LDL-C was associated with 40% increased risk of coronary heart disease (CHD) [8].

Based on this recent scientific evidence, the current updated guidelines on the management of dyslipidemia follow an aggressive approach towards lowering LDL‐C for the secondary prevention of acute coronary events. The European Society of Cardiology/European Atherosclerosis Society (ESC/EAS, 2019) guidelines give significant weightage to the measurement of LDL-C to evaluate the risk of cardiovascular events and to use LDL-C for monitoring treatment efficacy and patient compliance. These guidelines also provide importance on the role of other lipid fractions and takes into consideration the “Residual risk.” According to the American College of Cardiology/American Heart Association (ACC/AHA, 2018) guidelines, a patient with ACS must also have multiple risk factors or more than one previous ASCVD [9,10]. Both these international guidelines recommend drug therapy in patients with clinical ACS to lower the LDL-C level by at least ≥50%. For very high-risk ACS patients, the American guidelines recommend aiming for an LDL-C threshold of 70 mg/dL (1.8 mmol/L), while the European guidelines recommend an LDL-C reduction of at least 50% from baseline along with an LDL-C goal of <55 mg/dL (<1.4 mmol/L) for the secondary prevention [11].

Indians are observed to develop ACS at a younger age and have high mortality rates (over 50% of CHD mortality occurring in individuals aged >50 years) [12]. Hence, to prevent the ever-expanding epidemic of ACS, the Lipid Association of India (LAI, 2020) has recommended further stringent LDL-C goals, i.e., <50 mg/dL for very high-risk patients and ≤30 mg/dl for an extreme risk group of patients (having prior ASCVD event) [13].

In patients with ASCVD, secondary prevention with lipid-lowering medications is known to reduce the risk of clinical events and death. Han et al. conducted an analysis on 272,899 secondary prevention patients and identified that early treatment was associated with reductions in the risk of ASCVD hospitalization (p < 0.0001) [14]. Regardless of the LDL-C levels, statins are the first line of drug therapy in patients with high ASCVD. In a multicenter study involving 8168 patients, it was identified that half of the cohort were not on a statin prescription within 90 days of an ASCVD event [15]. In PURE study involving 33,423 subjects, it was found that the use of statin therapy among South Asian patients with CHD and stroke was only 5% [16]. In a recently conducted real-world population study (PINNACLE Registry), 84.5% of ASCVD patients reported not meeting the LDL-C goal of less than 70 mg/dL [17]. In a prospective, observational, multicenter cohort study conducted in India on 474 patients with an acute coronary event, it was identified that despite statin therapy, there was residual dyslipidemia [18]. In a study involving 5888 primary and secondary prevention CVD patients, despite the use of moderate-high intensity statins in combination with ezetimibe or PSCK9 inhibitors, only 54% of the patients achieved their risk-based 2016 ESC/EAS LDL-C goal [19]. To date, no major study has been conducted that could help in identifying the level of LDL-C control amongst Indian ACS patients, especially as per the revised LAI consensus and other global guidelines. Studies related to effective secondary prevention measures in patients with ASCVD are scarce, and the present study is being conducted to understand the current treatment patterns and attainment of guideline-directed LDL-C goals in real-world Indian settings.

## 2 Methods

In this retrospective, multicenter, observational study, paper-based records/Electronic Medical Records (EMRs) of patients having abnormal lipid levels after an index ACS event, receiving lipid-lowering therapies (LLTs) (such as statins or other drugs), and visiting outpatient department of Indian health care settings between January 2017–December 2020 were reviewed.

### 2.1 Study population

Patients >18 years with a history of an ACS event [One or more of the following: STEMI or left bundle branch block myocardial infarction (STEMI/LBBB MI), a NSTEMI or UA with or without percutaneous coronary intervention/coronary artery bypass graft] and having lipid profile data available at index date/15 days of the index date,12 months post index date and at least once at 3, 6, or 9 months after-index date ACS event were included in the study. Index date is the day on which ACS event has occurred [ACS event includes one or more of the following: ST-segment-elevation myocardial infarction or left bundle branch block myocardial infarction (STEMI/LBBB MI), a non-ST-segment-elevation myocardial infarction (NSTEMI) or UA]. Data on socio-demographic characteristics, medical history, medications, clinical presentation, diagnosis, and treatment were retrieved from the hospital records. However, patients with incomplete paper-based records/EMRs data were excluded from the study.

### 2.2 Study objectives and outcomes

The study was conducted in eleven different healthcare settings and populations in India to assess how current practice impacts LDL-C goal attainment. The primary objective of the study was to assess LDL-C control (<55 mg/dL) in patients with ACS with currently available treatments by determining the proportion of ACS patients achieving LDL-C goal of <55 mg/dL at different time points post index ACS event.

The study outcomes were evaluated at different time points, i.e., at 3, 6, 9, and 12 months after the index ACS event. The secondary outcomes of the study included assessing the proportion of ACS patients a) achieving LDL-C goal of <30, <50, <70, <100 mg/dL at different time points after index ACS event; b) who underwent Lipoprotein(a) testing and achieving Lp(a) levels (≤15 mg/dL or >15–30 mg/dL or >30–60 mg/dL or >60 mg/dL) during the study period; c) who exhibited a change in lipid profile parameters at different time points post index ACS event; d) receiving statins, exhibiting statin intolerance, receiving other LLTs, duration and modifications of LLTs at different time points as compared to baseline and; e) who reported AE during the study period. Additionally, the proportion of ACS patients with familial hypercholesteremia (FH) was also evaluated.

### 2.3 Statistical analysis

Data were analyzed using R studio 3.5.3. The descriptive analysis of the study data, including baseline characteristics, clinical characteristics, treatment details, etc., were presented and inferred. All the continuous variables (e.g., age, height, weight, etc.) were presented as mean ± SD, and the categorical variables (e.g., gender, location, etc.) were presented as frequency (or proportions). The proportion of ACS patients achieving a particular LDL-C goal at different time points post index ACS event was evaluated using the Chi-square test/Fisher's exact test/McNemar's test. Statistical significance was considered at *p* < 0.05.

### 2.4 Ethical statement

Patients’ confidentiality was maintained using anonymized and de-identified data at the source level. The data collection was performed as per the protocol and applicable ethical and regulatory guidelines, including the Declaration of Helsinki, Schedule Y, Indian GCP, and ICH-GCP. The study was approved by an independent ethics committee and institutional ethics committees. This study was registered in the Clinical Trial Registry of India (CTRI/2022/05/042446).

## 3 Results

### 3.1 Patient demographic details

A total of 575 patients from 11 centers across India were recruited for the study. Among 575 patients, 26 (4.52%) of the patients were below 35 years of age, 193 (33.57%) were between 36 and 50 years, 317 (55.13%) were 51–65 years, and 39 (6.78%) patients were 66 years and above. The data relating to familial hypercholesterolemia was unavailable for most patients (n = 480; 83.48%); three patients had familial hypercholesterolemia (0.52%). The clinical characteristics and comorbid conditions of the patients are presented in (Table 1).

**Table 1:** Baseline demographic and clinical characteristics of patients.

Parameters	Estimates
**N**	**575**
Female	136 (23.65%)
Mean Age (years)	52.92 ± 9.75
Mean Height (cms)	163.37 ± 6.62
Mean Weight (in kilograms)	71.20 ± 10.64
Mean BMI (kg/m^2^)	26.70 ± 3.97
**Education**	
Literate	392 (68.17%)
**Employment Status**	
Employed	337 (58.61%)
**Familial Hypercholesterolemia (FH)**	3 (0.52%)
**Diagnosis at discharge; n (%)**	
STEMI	339 (58.96%)
NSTEMI	141 (24.52%)
Unstable angina	95 (16.52%)
**Smoking status n (%)**	
Non-smoker	451 (78.43%)
Current	111 (19.3%)
Ex-smoker	13 (2.26%)
**Family history of CVD; n (%)**	
Yes	11 (1.91%)
No	207 (36%)
NA	357 (62.09%)
**History of prior CV events**	
Myocardial Infarction; n (%)	13 (2.26%)
Stroke; n (%)	3 (0.52%)
Congestive Heart Failure; n (%)	47 (8.17%)
Angina	13 (2.26%)
**Comorbid Conditions**	
**Type 2 Diabetes mellitus**	
Yes	164 (28.52%)
No	410 (71.30%)
NA	1 (0.17%)
Hypertension; n (%)	185 (32.17%)
CKD; n (%)	7 (1.22%)
Dyslipidaemia; n (%)	145 (25.22%)
**Other Comorbid Diseases; n (%)**	23 (4%)
Hypothyroidism	10 (43.48%)
Obesity	3 (13.04%)
Respiratory illness	5 (21.73%)
Bilateral Varicose Veins	1 (4.35%)
Dilated Cardiomyopathy	1 (4.35%)
Medical Management & EECP Therapy	1 (4.35%)
PSVT-Reverted with Adenosine	1 (4.35%)
Sclerosed & Calcified Aortic Valve	1 (4.35%)
Previous history of CV intervention; n (%)	10 (1.74%)
CABG; n (%)	3 (0.52%)
Thrombolytics; n (%)	1 (0.17%)

BMI- Body Mass Index, STEMI- ST Elevation Myocardial Infarction, NSTEMI- Non-ST segment Elevation Myocardial Infarction, T2DM- Type 2 diabetes mellitus, CKD-Chronic Kidney Disease, EECP- Enhanced external counter pulsation, PTB- Pulmonary tuberculosis, PSVT- Paroxysmal supraventricular tachycardia, CABG- Coronary Artery Bypass Graft, BP-Blood pressure, NA-Data not available.

### 3.2 Treatment details of patients involved in the study

The treatment modalities of the patients involved in the study are provided in Table 2. All the 575 patients involved in the study were on statins. Among the statins, atorvastatin was the most commonly prescribed statin. At the baseline, 373 patients (64.87%) received atorvastatin, which reduced to 292 (50.78%) at the end of 12 months. At the end of 12 months, none of the patients had dose escalation, and 18 (6.16%) had their dose reduced due to improved lipid profile, myalgia, and PI discretion. It was observed that 202 patients (35.13%) received rosuvastatin at baseline which increased to 282 (49.04%) at the end of 12 months. At the end of the study visit, 60 (21.28%) patients had the dose reduced, due to improved lipid profile, myalgia, and PI discretion. At the baseline 5 patients were on atorvastatin + ezetemibe, and 2 continued atorvastatin + ezetimibe at the end of 12 months. Six patients received rosuvastatin + ezetimibe at the baseline and 2 continued rosuvastatin + ezetimibe at the end of 12 months.

**Table 2:** Treatment details of patients involved in the study.

Parameters	Atorvastatin		Rosuvastatin		Ezetemibe		Fenofibrate	
	Baseline-n = 373 (64.87%)	12 months-n = 292 (50.78%)	Baseline n = 202 (35.13%)	12 months n = 282 (49.04%)	Baseline n = 11 (1.91%)	12 months n = 4 (0.69%)	Baseline n = 23 (4%)	12 months n = 10 (1.73%)
Dose of drug (mg)	10 mg- 2 (0.54%) 20 mg- 11 (2.95%) 40 mg- 157 (42.09%) 80 mg- 203 (54.42%)	10 mg- 5 (1.71%) 20 mg- 75 (25.68%) 40 mg- 133 (45.55%) 80 mg- 79 (27.05%)	10 mg- 6 (2.97%) 20 mg - 36 (17.82%) 30 mg- 0 (0%) 40 mg- 160 (79.21%) 80 mg- 0 (0%)	10 mg- 43 (7.48%) 20 mg- 134 (23.3%) 30 mg- 1 (0.17%) 40 mg- 102 (17.74%) 80 mg- 2 (0.35%)	10 mg- 11 (1.91%)	10 mg- 4 (0.69%)	145 mg- 19 (82.61%) 160 mg- 4 (17.39%)	145 mg- 10 (100%) 160 mg- 0
Dose Modifications (all dose reductions)	–	18 (6.16%)		60 (21.28%)				
Reasons for dose reductions: Deranged Lipid Profile Improved Lipid Profile Myalgia PI discretion Statin Intolerance		4 (22.22%)		9 (15%)				
		13 (72.22%)		48 (80%)				
		0 (0%)		1 (1.67%)				
		1 (5.56%)		2 (3.33%)				
		0 (0%)		0 (0%)				
Dose reduced to:		10 mg- 1 (5.56%)		10 mg- 22 (36.67%)				
		20 mg- 5 (27.78%)		20 mg- 31 (51.67%)				
		40 mg- 12 (66.67%)		40 mg- 7 (11.67%)				

### 3.3 Assessment of LDL-C targets during the study period

**Fig. 1:**
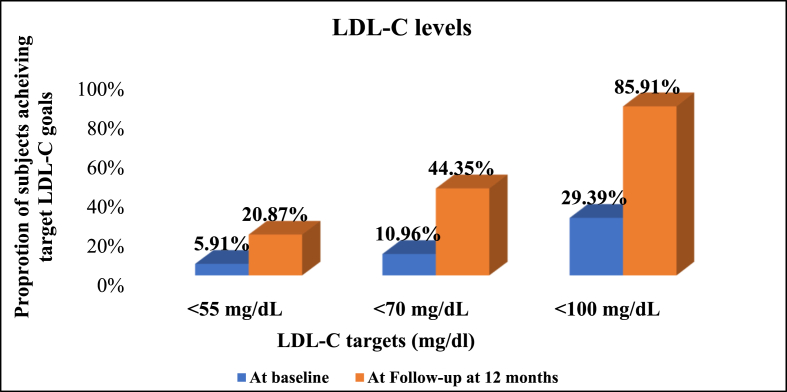
LDL-C levels at baseline and 12 months with different LDL target goals LDL-C- Low-density lipoprotein Cholesterol.

At the end of 12 months, 120 (20.87%) had LDL-C <55 mg/dl. The proportion of patients achieving LDL-C <55 mg/dl increased from 5.91% to 20.87% (post treatment). The proportion of patients attaining the different target LDL-C goals at the end of 12 months is provided in Fig. 1. Of the 575 patients analyzed, 55.65% could not reach LDL levels of <70 mg/dl one year after the event (Table 3).

**Table 3:** Assessment of LDL-C in patients with ACS during the study period.

Parameters	LDL Cholesterol			
	<30 mg/dL	<50 mg/dL	<70 mg/dL	<100 mg/dL
	N (%)	N (%)	N (%)	N (%)
Baseline				
Yes	5 (0.87%)	26 (4.52%)	63 (10.96%)	169 (29.39%)
No	570 (99.13%)	549 (95.48%)	512 (89.04%)	406 (70.61%)
FU at 1 year				
Yes	23 (4%)	94 (16.35%)	255 (44.35%)	494 (85.91%)
No	552 (96%)	481 (83.65%)	320 (55.65%)	81 (14.09%)
***p-value***	***0.001***	***<0.001***	***<0.001***	***<0.001***

FU- Follow-up.

Assessment of lipid profile and lipoprotein levels in ACS patients.

The change in the lipid profile parameters was evaluated by considering the mean levels of the respective parameters at the baseline and subsequent change during the follow-up visit. From baseline to one-year, total cholesterol reduced from 189.28 ± 52.48 mg/dl to 141.49 ± 34.24 mg/dl (p < 0.001), NHDL reduced from 143.84 ± 37.54 mg/dl to 100.48 ± 30.57 mg/dL (p < 0.001), LDL decreased from 122.64 ± 42.01 to 74.41 ± 26.45 mg/dl (p < 0.001), triglycerides reduced from 166.09 ± 62.80 mg/dl to 127.72 ± 51.17 mg/dl (p < 0.001). Also, it was observed that from baseline to one year, VLDL reduced from 34.34 ± 13.14 mg/dl to 27.28 ± 13.1 mg/dl (p < 0.001), Apo (B) levels reduced from 133.26 ± 10.25 mg/dl to 97.00 ± 17.96 mg/dl (p < 0.001), Lp (a) levels from 49.15 ± 15.03 mg/dl to 26.57 ± 16.23 mg/dl (p < 0.001). There was no significant increase in the mean HDL levels from baseline to the subsequent visits (Table 4).

**Table 4:** Assessment of lipid profile parameters in ACS patients.

Parameters	TC (mg/dL)	HDL (mg/dL)	NHDL (mg/dL)	LDL (mg/dL)	TG (mg/dL)	Apo (B) mg/dL	VLDL mg/dL	Lp(a) (mg/dL)
Baseline	189.28 ± 52.48	43.01 ± 25.4	143.84 ± 37.54	122.64 ± 42.01	166.09 ± 62.80	133.26 ± 10.25	34.34 ± 13.14	49.15 ± 15.03
FU at 1 year	141.49 ± 34.24	42.54 ± 20.53	100.48 ± 30.57	74.41 ± 26.45	127.72 ± 51.17	97.00 ± 17.96	27.28 ± 13.1	26.57 ± 16.23
***% Reduction***	***−25.25%***	***−1.09%***	***−30.14%***	***−39.33%***	***−23.10%***	***−27.21%***	***−20.56%***	***−45.94%***
***p-value***	***<0.001***	***0.674***	***<0.001***	***<0.001***	***<0.001***	***<0.001***	***<0.001***	***<0.001***

TC- Total Cholesterol, HDL- High-Density Lipoprotein, NHDL- Non-High-Density Lipoprotein, LDL-Low density Lipoprotein, TG-Triglycerides, Apo (B)- Apolipoprotein B, VLDL- Very Low-density lipoprotein, LP (a)- Lipoprotein a.

In real-world settings, the data related to lipoprotein(a) levels were available only for 121 patients. None of the patients were found to have Lp (a) ≤15 mg/dL at the baseline, and 32 (26.45%) had Lp (a) ≤15 mg/dL at the end of 12 months. The distribution of patients based on the Lp (a) levels is provided in Supplementary Table 1.

### 3.4 Assessment of tolerability of LLTs in ACS patients

The tolerability of LLTs, such as percentage of side effects reported by the patient and as captured by the clinician, are presented in Supplementary Table 2. It was observed that 6 patients had adverse events during the study period and out of which 4 were on rosuvastatin, and 2 on atorvastatin. Among the 6 patients, 4 had myalgia, and 2 had statin intolerance. All patients had a reduction in the dosage due to AEs and none discontinued the treatment due to adverse events.

## 4 Discussion

In this real-world evidence study, LDL-C control was evaluated in patients with ACS with currently available treatments. To the best of our knowledge, this is the first Indian study to evaluate the new target LDL-C levels in ASCVD patients after the index date/event. There was a statistically significant reduction in LDL-C levels from the baseline to the end of 12 months (p < 0.001). It was also observed that there was a statistically significant reduction in the mean levels of the other lipid profile parameters from the baseline. Among 575 patients, 94 had <50 mg/dL LDL-C and 481 had ≥50 mg/dL LDL-C at the end of 12 months.

In the current study, only 20.87% of patients managed to achieve the target LDL of <55 mg/dL, and 55.65% were unable to reach LDL-C levels of <70 mg/dl one year after the event. In a study conducted by Jaywant et al. it was observed that among the 118 patients with high LDL-C, 54 (45.8%) had LDL-C <70 mg/dl at the end of the study [18]. Kristensen et al. [20] reported that 40.7% and 39% attained the recommended LDL-C value of <1.8 mmol/L (<70 mg/dL) within the 6- and 12-months follow-up, respectively. In the DYSIS study [21] 48.2% of the patients did not meet the target LDL-C levels. In the CEPHEUS study [22] conducted in 35,121 participants from 29 countries, only 49.4% of the participants attained the recommended LDL-C levels. The 2019 ESC guidelines as well as the ACC 2022 Consensus reduced the LDL-C goal to <55 mg/dl from the previous <70 mg/dl based on mounting evidence of CV benefit with lower LDL levels that became possible with the advent of PCSK9is [9]. However, it seems this change in practice is slow on the uptake in India.

In this study, high levels of TC, TG, and LDL-C levels were observed at the baseline. Similarly, Patange et al. [23] identified that 37% of patients with ACS had high levels of TG, 31% of the patients had high TC, and 23% of patients had high levels of LDL-C (>130 mg/dl). In our study, most of the patients belonged to the 51–65 age group. Similarly, in a prospective observational study conducted in India [23], it was identified that among 100 ACS patients, the majority of them belonged to 51–60 years, and the prevalence of ACS was more among males. This ties in with the evidence that south Asians are at a higher risk of CV events as compared to their Caucasian counterparts [7].

Six patients had adverse events (4 were on rosuvastatin and 2 on atorvastatin) in the study requiring a reduction in the dosage, and none discontinued the treatment due to adverse events. However, in the REMAINS study [18], 7 patients developed serious adverse events.

LDL-C has been identified to be an independent risk factor in the development of ACS. In a retrospective cohort analysis [14], involving 272,899 secondary prevention patients, it was identified that patients with LDL-C levels >130 mg/dl experienced higher ASCVD hospitalization risks. Although appropriate treatment guidelines are available, there exists a significant treatment gap between treatment guidelines and clinical practice in the management of dyslipidemia in ACS. As LDL-C lowering is associated with more favorable cardiovascular outcomes [14], lipid management strategies need to be implemented to achieve guideline-recommended LDL-C targets for the management of patients with ACS. Though most patients were on high-intensity lipid-lowering therapy, more than half did not meet guideline-recommended LDL goals in one year. In the current study, out of 13.57% of the patients that underwent statin dose reductions, it was observed that most dose reductions were due to perceived improvement in lipid profile. However, only 20.87% of the patients achieved LDL-C <55 mg/dl at the end of one year, which shows a lack of awareness among the physicians to meet target LDL goals as per national and international guidelines. Although other lipid-lowering therapies are available and known to have proven outcomes in ACS, in the current study, they were not used.

There could be multiple possible reasons for the low achievement of LDL-C goals despite background lipid lowering therapy. In this study, the practice seen was of shifting from one statin to another, rather than escalating therapy to more efficient molecules. Patients started on Ezetemibe on a background of statin therapy were de-escalated by 1 year (4 patients on Ezetemibe instead of 11 initiated at baseline) and no patient was initiated on a PCSK9i throughout the study. This could suggest an unwillingness to escalate therapy either due to lack of awareness, high cost of additional therapy or inertia to change existing practice. Another aspect could be that doctors do not consider the LDL-C target of <55 mg/dl to be important for their very high-risk patients suggesting a gap in understanding the importance of these LDL-C goals. This reason would explain why >20% of the patients in this study had statin dose reductions at 1 year despite not reaching target goals. Cost of therapy would thus need to be compared to the cost of a recurrent CV event in case of inadequate LDL-C control. Another factor that usually goes unnoticed is treatment adherence of the patients. Poor adherence to prescribed therapy either due to patient laxity or adverse effects of the drug could also cause poor achievement of LDL-C targets. Although statin intolerance was not a factor for statin dose reductions in this study, there is a possibility that it was not documented or assessed as effects like muscle ache or body pain could be explained away by other reasons. It is therefore imperative that patient adherence is also assessed adequately to ensure optimum treatment and outcomes. Thus, innovator therapies with high efficacy and safety, and that can address patient adherence challenges could be useful in such cases.

As the clinical presentation of cardiovascular diseases is seen at a younger age in India and there is a suboptimal rate of guideline recommended management practices, more primary and secondary prevention strategies are required for the management of the disease. The existing gap between the clinical guidelines and the current practice need to be addressed by the collaborative efforts from the public private hospitals, newer interventions from the ministry of health and family welfare, and from the adequate support from the academic institutions [24,25].

The strength of the study includes adequate sample size, and as the study was conducted across India, this may be representative of all ACS patients across the country and may provide insights into the lipid abnormalities and residual dyslipidemia in Indian ACS patients after the index date/event. The study emphasizes that there is an underuse of available treatments (ezetimibe and PCSK9 inhibitors) in India. The present study has some limitations, including its retrospective design. Because the study is based on medical records, the data for follow-up from all patient visits to the doctor could not be retrieved, and the data on medication adherence to statins was unavailable. Despite the limitations, the study sheds light on the lipid levels in ACS patients during the index date and the follow-up period and the need to manage dyslipidemia in ACS with newer therapies.

## 5 Conclusions

In this real-world evidence study, there was a significant reduction in LDL-C levels post statin treatment in ACS patients. However, only 20.87% of the patients managed to reach the target of LDL-C of <55 mg/dl. Also, 55.65% of the patients could not reach LDL-C <70 mg/dl one year after the ACS event. As majority of the patients failed to achieve the guideline-recommended LDL-C levels after the ACS event there is a need for aggressive management with novel drugs that have a favorable safety and efficacy profile and that help patients attain the goal of <55 mg/dl as directed by the national and international guidelines. Further, large-scale prospective studies are warranted to validate the findings.

## Credit author statement

KS and PK contributed to the conception, design of the study, and interpretation and analysis of the data. MJ, RS, HP, AS, AJ, RG, KK, RJ, SM, AM, and NS contributed resources for the study. KS and PK drafted the manuscript. All authors critically revised the manuscript, approved the final manuscript, and agreed to be accountable for all aspects of the work, ensuring its integrity and accuracy.

## Funding

The study was funded by Novartis Healthcare Private Limited, India.

## Declaration of competing interest

Dr Kimi Shetty is the Medical Lead and Dr Pallavi Kawatra is the Franchise Medical Head for Novartis Healthcare Private Limited, India. All the other authors reported no other conflicts of interest for this work.
