# Supplementary material for: Evaluating LDL-C control in Indian acute coronary syndrome (ACS) patients- A retrospective real-world study LDL-C control in ACS
Source: Int J Cardiol Cardiovasc Risk Prev. 2023 Sep 16;19:200210. doi: 10.1016/j.ijcrp.2023.200210 (PMC10523158; doi:10.1016/j.ijcrp.2023.200210)
Supplement: Multimedia component 1 [file mmc1.docx]

**Supplementary Table 1: Assessment of Lipoprotein(a) or Lp(a) levels in ACS patients during the study period (N=121)**

| **Parameters** | **Lipoprotein(a) or Lp(a)** | | | |
| --- | --- | --- | --- | --- |
|  | **≤15 mg/dL** | **>15-30 mg/dL** | **>30-60 mg/dL** | **>60 mg/dL** |
|  | **N (%)** | **N (%)** | **N (%)** | **N (%)** |
| Baseline | 0 (0%) | 12 (9.92%) | 82 (67.77%) | 27 (22.31%) |
| FU within 12 months* | 9 (10.34%) | 53 (60.92%) | 25 (28.74%) | 0 (0%) |
| FU at 1 year | 32 (26.45%) | 59 (48.76%) | 19 (15.7%) | 11 (9.09%) |
| **Average of visits within 1 year i.e. (3 or 6, or 9 months)* | | | | |

FU-Follow-up

**Supplementary Table 2: Assessment of tolerability of LLTs in ACS patients**

| **Parameters** | **Total** | **Myalgia** | **Statin Intolerance** |
| --- | --- | --- | --- |
| No. of patients who reported adverse; n (%) | 6 (100%) | 4 (66.66%) | 2 (33.33%) |
| **Drug name & Dose** | | | |
| Rosuvastatin | 4 (66.66%) | 3 (75%) | 1 (25%) |
| 40 mg | 4 (100%) | 3 (100%) | 1 (100%) |
| Atorvastatin | 2 (33.33%) | 1 (50%) | 1 (50%) |
| 80 mg | 2 (100%) | 1 (100%) | 1 (100%) |
| **Association; n (%)** | | | |
| Certain | 2 (100%) | 0 (0%) | 2 (100%) |
| Probable | 4 (100%) | 4 (100%) | 0 (0%) |
| Possible | 0 (0%) | 0 (0%) | 0 (0%) |
| Unlikely | 0 (0%) | 0 (0%) | 0 (0%) |
| **Dose interruption (n, %)** | | | |
| No. of patients | 0 (0%) | 0 (0%) | 0 (0%) |
| Average duration of interrupted days | - | - | - |
| **Dose modification** | | | |
| Reduction | 6 (100%) | 4 (100%) | 2 (100%) |
| Escalation | 0 (0%) | 0 (0%) | 0 (0%) |
| **Reduction (n, %)** |  |  |  |
| Rosuvastatin | 4 (66.66%) | 3 (75%) | 1 (50%) |
| 10 mg | 1 (25%) | 0 (0%) | 1 (100%) |
| 20 mg | 3 (75%) | 3 (100%) | 0 (0%) |
| Atorvastatin | 2 (33.33%) | 1 (25%) | 1 (50%) |
| 40 mg | 2 (100%) | 1 (100%) | 1 (100%) |
| **Discontinuation (n, %)** |  |  |  |
| No. of patients | 0 (0%) | 0 (0%) | 0 (0%) |
| Average duration of discontinued days | - | - | - |
